# Structural Changes Induced by Daily Music Listening in the Recovering Brain after Middle Cerebral Artery Stroke: A Voxel-Based Morphometry Study

**DOI:** 10.3389/fnhum.2014.00245

**Published:** 2014-04-17

**Authors:** Teppo Särkämö, Pablo Ripollés, Henna Vepsäläinen, Taina Autti, Heli M. Silvennoinen, Eero Salli, Sari Laitinen, Anita Forsblom, Seppo Soinila, Antoni Rodríguez-Fornells

**Affiliations:** ^1^Cognitive Brain Research Unit, Cognitive Science, Institute of Behavioural Sciences, University of Helsinki, Helsinki, Finland; ^2^Finnish Centre of Interdisciplinary Music Research, University of Helsinki, Helsinki, Finland; ^3^Cognition and Brain Plasticity Group, Bellvitge Biomedical Research Institute (IDIBELL), L’Hospitalet de Llobregat, Barcelona, Spain; ^4^Department of Basic Psychology, University of Barcelona, Barcelona, Spain; ^5^Department of Radiology, HUS Medical Imaging Center, Helsinki University Central Hospital, University of Helsinki, Helsinki, Finland; ^6^Miina Sillanpää Foundation, Helsinki, Finland; ^7^Department of Music, University of Jyväskylä, Jyväskylä, Finland; ^8^Department of Neurology, Turku University Hospital, Turku, Finland; ^9^Institució Catalana de Recerca i Estudis Avançats (ICREA), Barcelona, Spain

**Keywords:** music, speech, stroke, magnetic resonance imaging, voxel-based morphometry, environmental enrichment, neuroplasticity, rehabilitation

## Abstract

Music is a highly complex and versatile stimulus for the brain that engages many temporal, frontal, parietal, cerebellar, and subcortical areas involved in auditory, cognitive, emotional, and motor processing. Regular musical activities have been shown to effectively enhance the structure and function of many brain areas, making music a potential tool also in neurological rehabilitation. In our previous randomized controlled study, we found that listening to music on a daily basis can improve cognitive recovery and improve mood after an acute middle cerebral artery stroke. Extending this study, a voxel-based morphometry (VBM) analysis utilizing cost function masking was performed on the acute and 6-month post-stroke stage structural magnetic resonance imaging data of the patients (*n* = 49) who either listened to their favorite music [music group (MG), *n* = 16] or verbal material [audio book group (ABG), *n* = 18] or did not receive any listening material [control group (CG), *n* = 15] during the 6-month recovery period. Although all groups showed significant gray matter volume (GMV) increases from the acute to the 6-month stage, there was a specific network of frontal areas [left and right superior frontal gyrus (SFG), right medial SFG] and limbic areas [left ventral/subgenual anterior cingulate cortex (SACC) and right ventral striatum (VS)] in patients with left hemisphere damage in which the GMV increases were larger in the MG than in the ABG and in the CG. Moreover, the GM reorganization in the frontal areas correlated with enhanced recovery of verbal memory, focused attention, and language skills, whereas the GM reorganization in the SACC correlated with reduced negative mood. This study adds on previous results, showing that music listening after stroke not only enhances behavioral recovery, but also induces fine-grained neuroanatomical changes in the recovering brain.

## Introduction

During the past 10 years, advanced magnetic resonance imaging (MRI) analysis methods, such as voxel-based morphometry (VBM) and diffusion tensor imaging (DTI), have provided novel information about the dynamics of the structural neuroplastic changes underlying spontaneous recovery and rehabilitation after stroke. Based on longitudinal VBM and DTI studies of stroke patients, the recovery of cognitive and motor deficits is associated with gray matter volume (GMV) changes in many frontal, temporal, cerebellar, and subcortical (e.g., hippocampus) areas (Grau-Olivares et al., 2010; Dang et al., 2013; Fan et al., 2013) as well as changes in the integrity of the white matter (WM) tracts connecting and projecting from these areas (Liang et al., 2008; van Meer et al., 2012; Thiebaut de Schotten et al., 2014). In longitudinal intervention studies, intensive motor rehabilitation using constraint-induced movement therapy (CIMT) has been shown to increase GMV in frontal and parietal sensory–motor areas and in the hippocampus (Gauthier et al., 2008) and intensive aphasia rehabilitation using constraint-induced language therapy (CILT) or melodic intonation therapy (MIT) has been observed to enhance the integrity of the WM tracks connecting frontal and temporal regions (arcuate fasciculus) in the left (Breier et al., 2011) and right (Schlaug et al., 2009) hemispheres, respectively. All in all, these findings suggest that both behavioral recovery and active rehabilitation after stroke are closely linked to fine-grained neuroanatomical changes in the recovering brain. However, very little is known about the wider potential effects of the *recovery environment* on structural brain plasticity after stroke in humans.

Converging evidence from both animal (Johansson, 2004; Nithianantharajah and Hannan, 2006) and human studies (Johansson, 2012; Janssen et al., 2014) indicates that an environmental enrichment (EE), which provides additional sensory, cognitive, motor, and/or social stimulation compared to a standard environment, plays an important role in enhancing behavioral recovery after an acute stroke. In addition, evidence from animal studies suggests that the post-stroke EE can induce a number of cellular and molecular neuroplastic effects in the brain, including increase in dendritic complexity (Biernaskie and Corbett, 2001; Johansson and Belichenko, 2002), neural stem and progenitor cells (Komitova et al., 2005; Matsumori et al., 2006), and neurotrophic and neural growth factor levels (Gobbo and O’Mara, 2004; Söderström et al., 2009), and that these changes are associated with better cognitive or motor recovery. Interestingly, especially a multisensory EE, which includes auditory, visual, and olfactory stimuli, has been found to be effective in improving cognitive and motor recovery and reducing lesion volume (Maegele et al., 2005a,b). Also evidence from developmental animal studies shows that a purely auditory EE, which contains complex sounds or music, can enhance the structure and function of the auditory cortex (Engineer et al., 2004; Bose et al., 2010) as well as improve learning and memory and upregulate various neurotransmitters (e.g., dopamine, glutamate) and neurotrophins associated with them (Sutoo and Akiyama, 2004; Angelucci et al., 2007; Nichols et al., 2007). Overall, these findings indicate that auditory enrichment can be beneficial for the brain and suggest that it could potentially contribute to better cognitive and neural recovery also after stroke.

In the human brain, music and speech constitute the two most complex and versatile auditory stimuli in terms of their acoustic richness and the breadth of the neural networks involved in their perception and learning (Zatorre, 2013). Neuroimaging studies of healthy subjects have demonstrated that music processing engages a vast bilateral network of temporal, frontal, parietal, cerebellar, and limbic/paralimbic areas associated with the perception of complex acoustic features (e.g., melody, rhythm), syntactic and semantic processing, attention and working memory, episodic and semantic memory, motor and rhythm processing, and experiencing emotions and reward (Blood and Zatorre, 2001; Janata et al., 2002a,b; Platel et al., 2003; Koelsch et al., 2004, 2005, 2006; Menon and Levitin, 2005; Bengtsson et al., 2009; Salimpoor et al., 2011, 2013; Alluri et al., 2012; Herdener et al., 2014; for recent reviews see, Koelsch, 2010, 2011; Zatorre, 2013). Evidence from VBM and DTI studies also indicates that frequent musical activities, such as playing an instrument or singing, can lead to long-term structural changes in the brain, especially in frontal, temporal, and parietal areas and in the WM pathways (e.g., corpus callosum, arcuate fasciculus) connecting them (Gaser and Schlaug, 2003; Hyde et al., 2009; Halwani et al., 2011; James et al., 2014). Improvements in attention and executive functioning have also been reported in healthy older adults after regular music playing activities, such as piano playing (Bugos et al., 2007), and one longitudinal study also highlighted the role of playing musical instruments and dancing as leisure activities associated with a reduced risk of developing dementia (Verghese et al., 2003). Regarding the potential rehabilitative use of music after stroke, results from recent clinical studies suggest that active music-based interventions that utilize singing (MIT) or instrument playing (music-supported therapy, MST), can be effective in improving speech and motor recovery through enhancing the functioning and connectivity of temporal auditory and frontal motor areas (Schlaug et al., 2008, 2009; Altenmüller et al., 2009; Rojo et al., 2011; Rodríguez-Fornells et al., 2012; Grau-Sánchez et al., 2013). Very little, however, is known about the potential neuroplastic changes induced by everyday musical activities, such as music listening, after stroke.

Previously, we performed a randomized controlled trial (RCT) concerning the potential rehabilitative effects of an enriched sound environment on stroke recovery. Sixty patients with an acute left (*n* = 29) or right (*n* = 31) hemisphere middle cerebral artery (MCA) brain infarction were randomized to a music group (MG) (daily listening to self-selected music), an audio book group (ABG) (daily listening to self-selected audio books), and a control group (CG) (standard care only) and their recovery was followed for 6 months using behavioral measures (neuropsychological tests and questionnaires on mood), an auditory magnetoencephalography (MEG) measurement, and structural MRI. Fifty-four patients completed the whole 6-month follow-up. Behavioral results showed that verbal memory and focused attention improved more in the MG than in the ABG or CG after the intervention period at the 3-month follow-up and also remained better at the longitudinal 6-month follow-up (Särkämö et al., 2008), suggesting that regular music listening enhanced cognitive recovery. Compared to the CG, the MG also experienced less depressed and confused mood at the 3-month follow-up (Särkämö et al., 2008). MEG results showed that the mismatch negativity (MMN) response to frequency changes strengthened more in the MG and ABG compared to the CG at the 6-month follow-up, indicating that regular exposure to both music and speech enhanced early auditory encoding in the recovering brain (Särkämö et al., 2010a).

In the present study, our aim was to determine with a VBM analysis of the longitudinal structural MRI data (baseline acute stage and 6-month stage) from the same patient sample whether daily music listening could also lead to structural GM and WM reorganization in the brain and if this change would also be related to the previously found positive effects of music on cognitive and emotional recovery after stroke.

## Materials and Methods

### Subjects and study design

Sixty stroke patients were recruited during 2004–2006 from the Department of Neurology of the Helsinki University Central Hospital (HUCH). All patients had an acute ischemic MCA stroke in the left (*n* = 29) or right (*n* = 31) temporal, frontal, parietal, or subcortical brain regions. Additional inclusion criteria were: no prior neurological/psychiatric disease, drug/alcohol abuse, or hearing deficit; right-handed; ≤75 years old; Finnish-speaking; and able to co-operate. Recruited patients were randomly assigned to one of three groups (*n* = 20 in each): an MG, an ABG, or a CG. Randomization was performed with a random number generator by a researcher not involved in the patient enrollment. The study was approved by the HUCH Ethics Committee, and all patients signed an informed consent. All patients received standard treatment for stroke in terms of medical care and rehabilitation.

During the follow-up, the patients underwent a neuropsychological assessment (including cognitive tests and questionnaires) and an auditory MEG measurement 1 week (baseline), 3 months, and 6 months post-stroke, and a structural MRI within 2 weeks of the stroke onset and 6 months post-stroke. Details regarding the methodology and results of the neuropsychological assessments and the MEG experiment are available in the previous published articles (Särkämö et al., 2008, 2010a).

Of the 60 patients originally recruited into the study, 55 completed the study up to the 3-month stage and 54 up to the 6-month stage. For the purpose of the longitudinal VBM analyses, appropriate MRI data were unavailable in three patients and the image quality was insufficient in two further patients. Thus, data from 49 patients were used in the present study. Demographic and clinical characteristics as well as the musical and linguistic activities of the patients are shown in Tables 1 and 2, presented separately for the patients with left hemisphere damage (LHD, *n* = 23) and right hemisphere damage (RHD, *n* = 26). There were no significant differences between the MG, ACG, and CG on any demographic or clinical variables, prior musical or linguistic activities, or in other rehabilitation received during the 6-month follow-up whereas the frequency of listening to music and audio books differed highly significantly between the groups both at the 3-month and the 6-month stage. However, there were no statistically significant differences between the MG and ABG on how many hours per day the patients listened to the provided material (music in the MG, audio books in the ABG) on average, although within the RHD patients the daily listening amounts were slightly higher in the MG than in the ABG. Overall, these results indicate that the groups were comparable and that the intervention protocol worked well.

**Table 1:** **Demographic and clinical characteristics of the patients (*n* = 49)**.

	Left hemisphere damage (*n* = 23)				Right hemisphere damage (*n* = 26)			
	MG (*n* = 7)	ABG (*n* = 8)	CG (*n* = 8)	*p*-value	MG (*n* = 9)	ABG (*n* = 10)	CG (*n* = 7)	*p*-value
**DEMOGRAPHICAL CHARACTERISTICS**								
Age (years)	55.3 (11.0)	57.9 (7.4)	60.0 (8.9)	0.615 (F)	59.6 (7.9)	59.4 (9.0)	63.4 (4.7)	0.519 (F)
Gender (male/female)	3/4	6/2	5/3	0.439 (χ^2^)	6/3	2/8	3/4	0.111 (χ^2^)
Education (years)	10.9 (4.4)	12.9 (3.1)	9.9 (3.4)	0.262 (F)	11.1 (4.5)	11.3 (2.9)	9.3 (3.9)	0.518 (F)
**CLINICAL CHARACTERISTICS**								
Time from stroke onset to acute MRI (days)	7.0 (3.2)	7.6 (3.7)	7.6 (2.8)	0.922 (F)	6.9 (1.6)	9.1 (3.4)	8.2 (4.3)	0.366 (F)
Time from stroke onset to 6-month MRI (days)	184.4 (6.2)	184.6 (15.9)	192.4 (16.2)	0.449 (F)	189.0 (5.9)	182.7 (8.8)	193.9 (22.0)	0.228 (F)
Hemiparesis (yes/no)	5/2	4/4	3/5	0.409 (χ^2^)	9/0	10/0	7/0	–
Aphasia (yes/no)	4/3	6/2	6/2	0.701 (χ^2^)				
Lesion size (max. diameter in cm)	47.0 (17.8)	39.7 (18.8)	52.5 (17.6)	0.384 (F)	57.3 (28.9)	58.5 (20.5)	62.1 (25.3)	0.924 (F)
**OTHER REHABILITATION DURING THE 6-MONTH FOLLOW-UP^a^**								
Physical therapy	19.7 (30.5)	11.3 (30.0)	4.0 (9.4)	0.732 (K)	26.9 (41.6)	30.2 (37.7)	20.1 (26.5)	0.991 (K)
Occupational therapy	14.3 (22.7)	0.4 (0.8)	8.0 (20.3)	0.627 (K)	9.8 (12.9)	10.0 (14.7)	5.9 (6.4)	0.886 (K)
Speech therapy	15.3 (20.5)	3.7 (9.4)	10.9 (11.5)	0.340 (K)	15.3 (20.5)	3.7 (9.4)	10.9 (11.5)	0.340 (K)
Neuropsychological rehabilitation	4.0 (10.9)	2.3 (4.3)	1.0 (1.9)	0.851 (K)	2.9 (5.1)	2.5 (4.9)	0.7 (1.0)	0.976 (K)

*Data are mean (SD) unless otherwise stated. MG, music group; ABG, audio book group; CG, control group; *F*, one-way ANOVA; χ^2^, chi-square test (likelihood ratio); K, Kruskal–Wallis test*.

*^a^Number of therapy sessions*.

**Table 2:** **Musical and linguistic activities of the patients (*n* = 49)**.

	Left hemisphere damage (*n* = 23)				Right hemisphere damage (*n* = 26)			
	MG (*n* = 7)	ABG (*n* = 8)	CG (*n* = 8)	*p*-value	MG (*n* = 9)	ABG (*n* = 10)	CG (*n* = 7)	*p*-value
**BEFORE STROKE**								
Listening to music				0.592 (K)				0.617 (K)
Never	0 (0)	0 (0)	0 (0)		0 (0)	0 (0)	1 (14.3)	
Rarely	1 (14.3)	1 (12.5)	2 (25)		2 (22.2)	2 (20)	0 (0)	
Once a month	0 (0)	0 (0)	1 (12.5)		0 (0)	1 (10)	0 (0)	
Once a week	1 (14.3)	3 (37.5)	1 (12.5)		0 (0)	1 (10)	2 (28.6)	
2–3 Times a week	3 (42.9)	4 (50)	2 (25)		2 (22.2)	3 (30)	0 (0)	
Daily	2 (28.6)	0 (0)	2 (25)		5 (55.6)	3 (30)	4 (57.1)	
Listening to radio				0.991 (K)				0.163 (K)
Never	0 (0)	0 (0)	0 (0)		0 (0)	0 (0)	0 (0)	
Rarely	1 (14.3)	0 (0)	1 (12.5)		0 (0)	1 (10)	0 (0)	
Once a month	0 (0)	0 (0)	1 (12.5)		0 (0)	1 (10)	0 (0)	
Once a week	1 (14.3)	2 (25)	0 (0)		0 (0)	1 (10)	1 (14.3)	
2–3 Times a week	0 (0)	1 (12.5)	0 (0)		1 (11.1)	1 (10)	3 (42.9)	
Daily	5 (71.4)	5 (62.5)	6 (75)		8 (88.9)	6 (60)	3 (42.9)	
Reading				0.686 (K)				0.220 (K)
Never	0 (0)	0 (0)	0 (0)		0 (0)	0 (0)	0 (0)	
Rarely	0 (0)	0 (0)	0 (0)		0 (0)	0 (0)	0 (0)	
Once a month	0 (0)	0 (0)	1 (12.5)		1 (11.1)	0 (0)	0 (0)	
Once a week	0 (0)	1 (12.5)	1 (12.5)		4 (44.4)	4 (40)	0 (0)	
2–3 Times a week	3 (42.9)	1 (12.5)	1 (12.5)		2 (22.2)	2 (20)	3 (42.9)	
Daily	4 (57.1)	4 (50)	5 (62.5)		2 (22.2)	4 (40)	4 (57.1)	
**DURING THE FIRST 3 MONTHS POST-STROKE**								
Listening to music				0.003 (K)				0.001 (K)
Data missing	1 (14.3)	0 (0)	0 (0)		1 (11.1)	0 (0)	0 (0)	
Never	0 (0)	6 (75)	5 (62.5)		0 (0)	2 (20)	3 (42.9)	
Rarely	0 (0)	1 (12.5)	1 (12.5)		0 (0)	3 (30)	1 (14.3)	
Once a month	0 (0)	0 (0)	0 (0)		0 (0)	1 (10)	0 (0)	
Once a week	0 (0)	0 (0)	0 (0)		0 (0)	1 (10)	0 (0)	
2–3 Times a week	0 (0)	0 (0)	1 (12.5)		0 (0)	2 (20)	2 (28.6)	
Daily	6 (85.7)	1 (12.5)	1 (12.5)		8 (88.9)	1 (10)	1 (14.3)	
Listening to audio books				<0.001 (K)				<0.001 (K)
Data missing	1 (14.3)	0 (0)	0 (0)		0 (0)	0 (0)	0 (0)	
Never	5 (71.4)	0 (0)	6 (75)		9 (100)	1 (10)	6 (85.7)	
Rarely	1 (14.3)	0 (0)	2 (25)		0 (0)	0 (0)	0 (0)	
Once a month	0 (0)	0 (0)	0 (0)		0 (0)	0 (0)	0 (0)	
Once a week	0 (0)	1 (12.5)	0 (0)		0 (0)	0 (0)	0 (0)	
2–3 Times a week	0 (0)	2 (25)	0 (0)		0 (0)	0 (0)	0 (0)	
Daily	0 (0)	5 (62.5)	0 (0)		0 (0)	9 (90)	1 (14.3)	
Hours per day listening to group material^a^	1.6 (0.7)	1.3 (0.5)	–	0.484 (T)	2.1 (0.6)	1.4 (0.7)	–	0.076 (T)
**DURING 3–6 MONTHS POST-STROKE**								
Listening to music				0.010 (K)				0.018 (K)
Data missing	0 (0)	0 (0)	1 (12.5)		1 (11.1)	0 (0)	0 (0)	
Never	0 (0)	0 (0)	2 (25)		0 (0)	2 (20)	2 (28.6)	
Rarely	0 (0)	1 (12.5)	2 (25)		0 (0)	1 (10)	1 (14.3)	
Once a month	0 (0)	2 (25)	0 (0)		0 (0)	3 (30)	1 (14.3)	
Once a week	0 (0)	1 (12.5)	2 (25)		1 (11.1)	2 (20)	0 (0)	
2–3 Times a week	3 (42.9)	0 (0)	1 (12.5)		3 (33.3)	1 (10)	2 (28.6)	
Daily	4 (57.1)	4 (50)	0 (0)		4 (44.4)	1 (10)	1 (14.3)	
Listening to audio books				<0.001 (K)				0.004 (K)
Data missing	0 (0)	0 (0)	1 (12.5)		0 (0)	0 (0)	0 (0)	
Never	6 (85.7)	0 (0)	5 (62.5)		8 (88.9)	2 (20)	6 (85.7)	
Rarely	1 (14.3)	0 (0)	2 (25)		0 (0)	1 (10)	0 (0)	
Once a month	0 (0)	2 (25)	0 (0)		0 (0)	0 (0)	0 (0)	
Once a week	0 (0)	0 (0)	0 (0)		0 (0)	2 (20)	0 (0)	
2–3 Times a week	0 (0)	3 (37.5)	0 (0)		1 (11.1)	1 (10)	1 (14.3)	
Daily	0 (0)	3 (37.5)	0 (0)		0 (0)	4 (40)	0 (0)	

*Data shown as frequency (percentage) unless otherwise stated. MG, music group; ABG, audio book group; CG, control group; K, Kruskal–Wallis test; T, *t*-test*.

*^a^Music listening in the MG and audio book listening in the ABG [data are mean (SD)]*/

### Intervention

As soon as possible after their enrollment to the study (mean 8.8 days post-stroke, range 3–21 days), the MG and ABG patients were individually contacted by a music therapist. In the MG, the therapist provided the patients with portable CD players and CDs of their own favorite music in any musical genre (mostly popular music with lyrics but also jazz, folk, or classical music). Similarly, the therapist provided the ABG with portable players and self-selected narrated audio books. The patients were trained in using the players and were instructed to listen to the material by themselves daily (for a minimum of 1 h per day) for the following 2 months in addition to standard care and rehabilitation. After this intervention period (3-month stage), they were encouraged to continue listening to the material on their own. In order to ensure that the patients were able to engage in the listening protocol, the therapist kept close weekly contact with the patients and the nurses and/or relatives of the patients were asked to help. Frequency of listening was verified from the listening diaries, which the patients kept during the intervention period and from questionnaires at the 3- and 6-month stages. The CG was not given any listening material and received only the standard care and rehabilitation during the follow-up.

### MRI data acquisition

Structural MRI was performed within 2 weeks of stroke onset and 6 months post-stroke using the 1.5 T Siemens Vision scanner of the HUCH Department of Radiology. Clinically, the MRI was used by two experienced neuroradiologists (authors Taina Autti and Heli M. Silvennoinen) to verify the stroke diagnosis and to evaluate the size and location of the lesion. The MRI sequence included a 3D set of high-resolution T1 images (*T*_E_ = 3.68 ms, *T*_R_ = 1900 ms, *T*_I_ = 1100 ms, flip angle 15°, isotropic voxel size of 1 mm^3^), which were used in the present VBM analysis. In addition, also a smaller set of fluid-attenuated inversion recovery (2D FLAIR) images, which are sensitive to acute infarcts, were acquired and used in accurately locating the lesion area, especially in the acute stage.

### Voxel-based morphometry analysis

Morphometric analysis was carried out using VBM (Ashburner and Friston, 2000) and Statistical Parametric Mapping software (SPM8; The Welcome Department of Imaging Neuroscience, London) under MATLAB 7.8.0 (The MathWorks Inc., Natick, MA, USA). The normalization of brain images is a prerequisite in any multi-subject voxel-wise MRI data analysis and especially important when dealing with abnormal brains. In order to achieve an accurate segmentation and normalization of lesioned GM and WM tissue, Unified Segmentation (Ashburner and Friston, 2005) with medium regularization and cost function masking (CFM) was applied to the structural T1-weighted images of each subject (Brett et al., 2001). The cost function masks were defined by manually depicting for each patient at each time (acute and 6-month stage) binary lesion masks of the lesioned tissue using the MRIcron software package^1^ (Rorden and Brett, 2000). This technique has been widely used with patients suffering from stroke (Crinion et al., 2007; Andersen et al., 2010; Ripollés et al., 2012), achieving optimal normalization with no post-registration lesion shrinkage or out-of-brain distortion (Ripollés et al., 2012). During normalization, the GM and WM images were modulated in order to preserve the total amount of the signal. The resulting normalized GM and WM tissue probability maps were smoothed by using an isotropic spatial filter (FWHM = 6 mm) to reduce residual inter-individual variability.

All normalized and smoothed GM and WM images were further analyzed in order to compare the differences in the GMV or white matter volume (WMV). Because the processing of music and speech are generally known to involve the left and right hemispheres to a different degree (e.g., Zatorre et al., 2002; Tervaniemi and Hugdahl, 2003) and they are therefore differentially affected by lesion laterality, we performed separate analyses for the LHD patients (MG: *n* = 7, ABG: *n* = 8, CG: *n* = 8) and the RHD patients (MG: *n* = 9, ABG: *n* = 10, CG: *n* = 7). Thus, four separate mixed-design analysis of variance (ANOVA) models (GMV–LHD, GMV–RHD, WMV–LHD, WMV–RHD) were built with Group (MG/ABG/CG) as a between-subjects variable and Time (acute stage/6-month stage) as a within-subjects variable (thereby ensuring that each subject acted as its own control). Total intracranial volume (TIV) was included as a nuisance variable in order to correct for global differences for head size. Three different Group × Time interactions were calculated: MG > CG and ABG, ABG > CG and MG, CG > MG and ABG. In other words, we tested if the increments in post–pre GMV in one group (e.g., MG) were greater than in the other two groups (e.g., CG and ABG). In addition, *post hoc* paired *t*-tests were planned to check the direction of the effect of Time within each Group (6 months > acute). It has been suggested that combined intensity and cluster size thresholds such as *p* < 0.005 with a 10 voxel extent produce a desirable balance between Type I and Type II errors (Lieberman and Cunningham, 2009). Taking a slightly more stringent approach, the results are reported in tables at *p* < 0.001 (uncorrected threshold) with a cluster size of ≥50 voxels of spatial extent. For the sake of visual clarity, results are shown in figures at *p* < 0.01 (uncorrected threshold), although only clusters reported in the tables are labeled and commented throughout the text. Anatomical and cytoarchitectonical areas were identified using the Automated Anatomical Labeling (Tzourio-Mazoyer et al., 2002) and the Talairach Daemon database atlases (Lancaster et al., 2000) included in the xjView toolbox^2^.

Finally, for any cluster of voxels where a significant Group × Time interaction was found, mean GMV or WMV increase (6 months − acute stage) was calculated for each patient and correlated with behavioral changes (also 6 months − acute stage) in cognitive tests and mood scales. For the cognitive measures, changes in the summary scores of the tests measuring the following cognitive domains were included: verbal memory, short-term and working memory, language skills, visuospatial cognition, executive functions, focused attention (correct responses and reactions times), and sustained attention (correct responses and reactions times; for details, see Särkämö et al., 2008). Similarly, for the mood measures, changes in the eight Profile of Mood States (POMS) scales (tension, depression, irritability, vigor, fatigue, inertia, confusion, and forgetfulness) were included (for details, see Särkämö et al., 2008).

## Results

### Gray and white matter volume changes during recovery

**Figure 1:**
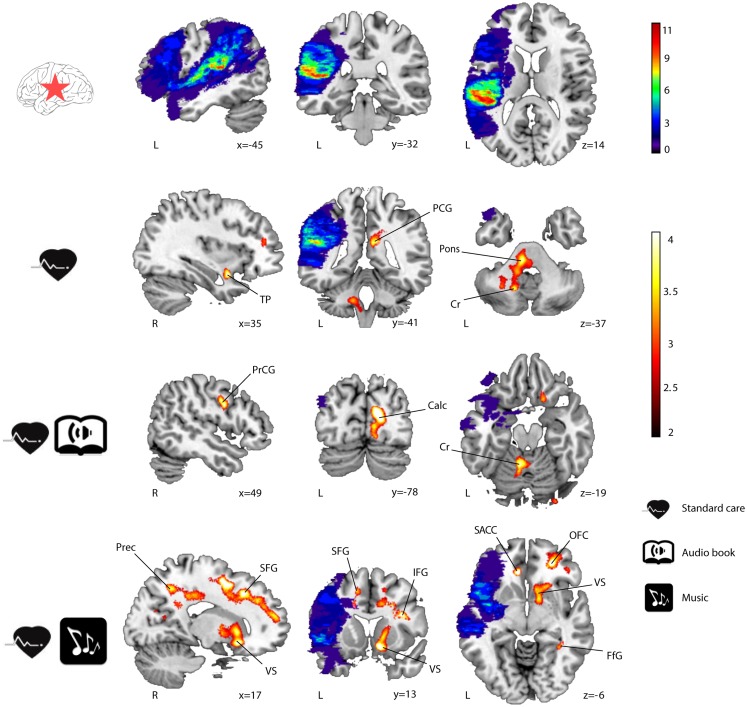
**GMV increases (6-month − acute) in LHD patients (*n* = 23)**. Lesion overlap indicating the number of patients with damage at a particular voxel and GMV increases within the three groups are shown in blue–green–red and red–yellow, respectively. Neurological convention is used. Results are shown at *p* < 0.01 (uncorrected) with ≥50 voxels of spatial extent and overlaid over a canonical template with MNI coordinates at the bottom right of each slice. Only clusters surviving a *p* < 0.001 threshold are labeled (see also Table 3). TP, temporal pole; PCG, posterior cingulate gyrus; Cr, cerebellum; PrCG, precentral gyrus; Calc, calcarine; Prec, precuneus; SFG, superior frontal gyrus; VS, ventral striatum; IFG, inferior frontal gyrus; SACC, ventral/subgenual anterior cingulate cortex; OFC, orbitofrontal cortex; FfG, fusiform gyrus; L, left hemisphere; R, right hemisphere.

**Figure 2:**
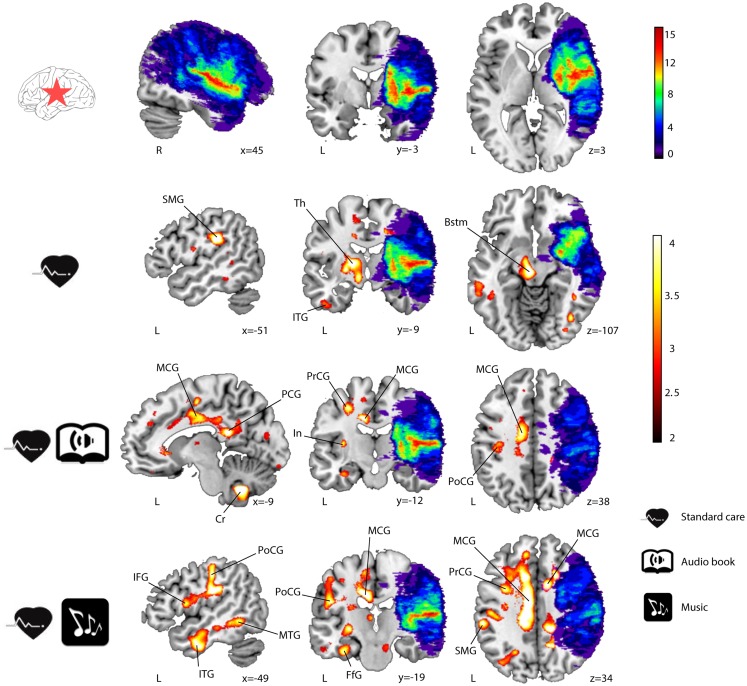
**GMV increases (6-month − acute) in RHD patients (*n* = 26)**. Lesion overlap indicating the number of patients with damage at a particular voxel and GMV increases within the three groups are shown in blue–green–red and red–yellow, respectively. Neurological convention is used. Results are shown at *p* < 0.01 (uncorrected) with ≥50 voxels of spatial extent and overlaid over a canonical template with MNI coordinates at the bottom right of each slice. Only clusters surviving a *p* < 0.001 threshold are labeled (see also Table 4). SMG, supramarginal gyrus; Th, thalamus; ITG, inferior temporal gyrus; Bstm, brainstem; MCG, middle cingulate gyrus; PCG, posterior cingulate gyrus; Cr, cerebellum; PrCG, precentral gyrus; In, insula; PoCG, postcentral gyrus; IFG, inferior frontal gyrus; MTG, middle temporal gyrus; FfG, fusiform gyrus; L, left hemisphere; R, right hemisphere.

Significant GMV increases were found post-intervention (6 months − acute) for all three groups of LHD patients (see Table 3; Figure 1) and RHD patients (see Table 4; Figure 2). Areas identified were mostly located in the temporal, frontal, motor, limbic, and cerebellar brain regions, especially in the contralesional hemisphere, with the largest and most extensive volume increases occurring in the MG.

**Table 3:** **GMV increases (6-month − acute) in LHD patients (*n* = 23)**.

	Anatomical area	MNI coordinates	Cluster size	*t*-value
CG	Left cerebellum	−14 −59 −40	189	4.75
	Right temporal pole (BA 38)	34 5 −20	111	4.46
	Right cerebellum	14 −59 −42	59	4.27
	Left pons	−7 −29 −35	203	4.08
	Right posterior cingulate gyrus	12 −40 24	83	3.96
ABG	Right calcarine/cuneus (BA 17, 18)	17 −77 12	1084	5.14
	Left cerebellum	−7 −53 −17	166	4.75
	Right pons	−18 −35 −42	85	4.71
	Right calcarine (BA 17)	9 −82 4	239	4.66
	Right precentral gyrus (BA 6)	47 0 31	110	4.18
MG	Left ventral/subgenual anterior cingulate cortex (BA 10)	−10 34 −3	185	5.75
	Right superior frontal gyrus (BA 32, 6)	19 4 51	588	5.54
	Right middle frontal gyrus (BA 32, 9)	21 24 39	1367	5.39
	Right inferior frontal gyrus	31 13 21	153	5.35
	Right ventral striatum	12 15 −11	484	4.85
	Right fusiform gyrus (BA 19)	32 −45 −9	130	4.82
	Right orbitofrontal cortex (BA 11)	28 44 −6	264	4.80
	Right superior frontal gyrus (BA 10)	22 51 2	490	4.74
	Right superior medial frontal gyrus (BA 8)	8 32 51	76	4.67
	Right precuneus (BA 7)	19 −53 46	166	4.60
	Right posterior cingulate gyrus	14 −40 25	471	4.46
	Right ventral striatum/globus pallidum	14 6 −2	252	4.43
	Left supplementary motor area	−11 10 50	54	4.07

*Results are reported at a *p* < 0.001 (uncorrected threshold) with 50 voxels of spatial extent. CG, control group; ABG, audio book group; MG, music group; BA, Brodmann area*.

**Table 4:** **GMV increases (6-month − acute) in RHD patients (*n* = 26)**.

	Anatomical area	MNI coordinates	Cluster size	*t*-value
CG	Left supramarginal gyrus (BA 40)	−44 −28 28	1597	6.13
	Left thalamus	−15 −6 6	946	5.97
	Left brainstem	−4 −20 −10	695	5.27
	Left inferior temporal lobe (BA 20, 21)	−44 −5 −35	208	4.42
	Right cerebellum	14 −42 −25	93	4.19
	Left sup./mid. occipital gyrus	−21 −86 12	405	4.18
	Left cuneus (BA 18)	−5 −85 22	340	4.18
	Left orbitofrontal cortex (BA 47)	−16 25 −21	85	3.97
ABG	Left cerebellum	−9 −52 −40	874	6.42
	Left posterior cingulum	−12 −43 21	322	5.85
	Right posterior cingulum	20 −43 26	79	4.83
	Left middle cingulum	−12 −11 35	546	4.78
	Right orbitofrontal cortex (BA 10)	15 53 1	185	4.66
	Right cerebellum	16 −71 −51	268	4.64
	Left thalamus	−15 −6 11	105	4.40
	Left insula	−33 −14 10	61	4.21
	Left precuneus	−20 −50 14	59	4.19
	Left precentral gyrus (BA 6)	−29 −11 45	102	4.18
	Left postcentral gyrus (BA 4)	−43 −17 51	169	4.13
MG	Right precuneus (BA 31)	16 −45 21	1544	6.35
	Left post/middle/ant cingulate gyrus; left sup./mid. frontal gyrus; left supp. motor area; left inferior frontal gyrus; *pars triangularis* (BA 32, 31, 24, 9)	−10 −12 34	11173	6.16
	Left supramarginal gyrus; left postcentral gyrus (BA 40)	−48 −23 27	1472	6.09
	Left inferior frontal gyrus; left precentral gyrus (BA 6)	−42 9 17	799	5.92
	Right middle cingulate gyrus (BA 24)	18 30 26	970	4.90
	Left orbitofrontal cortex (BA 47, 11)	−25 23 −16	1661	4.85
	Left inf./mid. temporal gyrus (BA 20)	−46 −5 −31	717	4.56
	Left fusiform gyrus	−34 −17 −27	266	4.42
	Left insula	−34 −12 −7	387	4.39
	Left parahippocampal gyrus	26 −65 −38	191	4.21
	Right cerebellum	11 31 −11	280	4.18
	Right anterior cingulate (BA 32)	19 43 4	72	4.14
	Left precentral gyrus	−34 5 35	188	4.12
	Left middle temporal gyrus	−56 −46 −8	372	4.06
	Right fusiform gyrus	36 −42 −16	246	4.05

*Results are reported at a *p* < 0.001 (uncorrected threshold) with 50 voxels of spatial extent. CG, control group; ABG, audio book group; MG, music group; BA, Brodmann area*.

**Figure 3:**
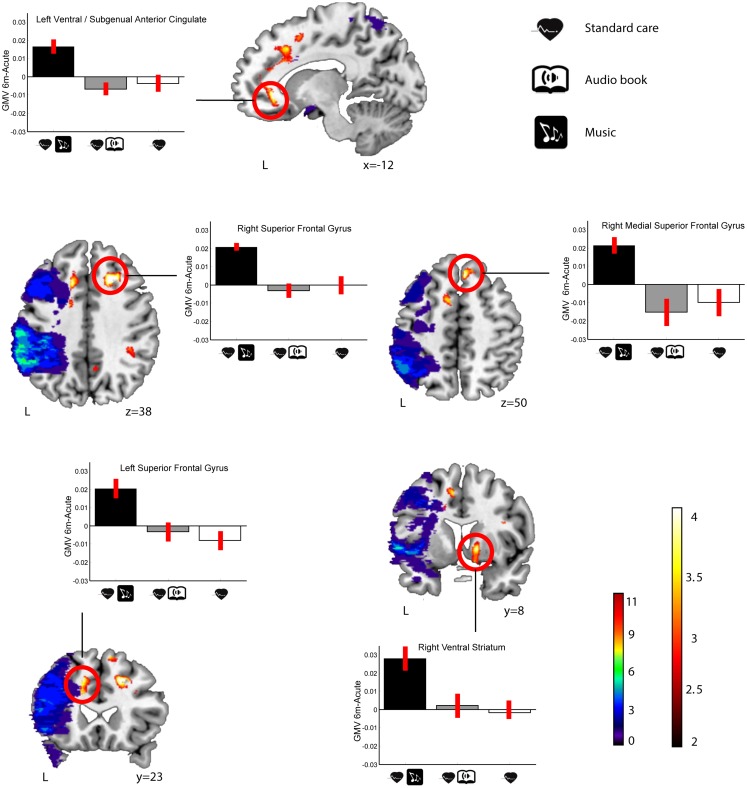
**GMV increases (6-month − acute) in the MG compared to the ABG and CG (LHD patients)**. Blue–green–red: lesion overlap indicating the number of patients showing damage at a particular voxel. Red–yellow: GMV increases for the MG compared to the ABG and CG (Group × Time interaction, MG > ABG and CG contrast). Bar graphs indicate GMV increases (mean ± SEM) for each of the clusters showing an interaction effect (white: CG, gray: ABG, black: MG). Neurological convention is used. Results are shown at *p* < 0.01 (uncorrected) with ≥50 voxels of spatial extent and overlaid over a canonical template with MNI coordinates at the bottom right of each slice (see also Table 5). L, left hemisphere.

In LHD patients, significant Group × Time interactions in GMV were found for the MG > ABG and CG contrast (see Table 5; Figure 3) in five different clusters: three in frontal areas [left and right superior frontal gyrus (SFG) and right medial SFG] and two in limbic areas [left ventral/subgenual anterior cingulate cortex (SACC) and right ventral striatum (VS) / globus pallidum). The reversed contrasts (ABG > CG and MG, CG > MG and ABG) did not yield any significant regions.

**Table 5:** **GMV increases (6-month − acute) in the MG compared to the ABG and CG (LHD patients)**.

Anatomical area	MNI coordinates	Cluster size	*t*-value
Right superior frontal gyrus (BA 32)	22 25 38	347	4.88
Left ventral/subgenual anterior cingulate cortex (BA 10)	−10 37 −4	53	4.51
Right medial superior frontal gyrus (BA 8)	7 32 51	57	4.31
Right ventral striatum/globus pallidum	14 5 −3	57	4.03
Left superior frontal gyrus (BA 32)	−13 21 38	51	4.01

*Time × Group interaction (MG > ABG and CG) at a *p* < 0.001 (uncorrected threshold) with 50 voxels of spatial extent. BA, Brodmann area*.

**Figure 4:**
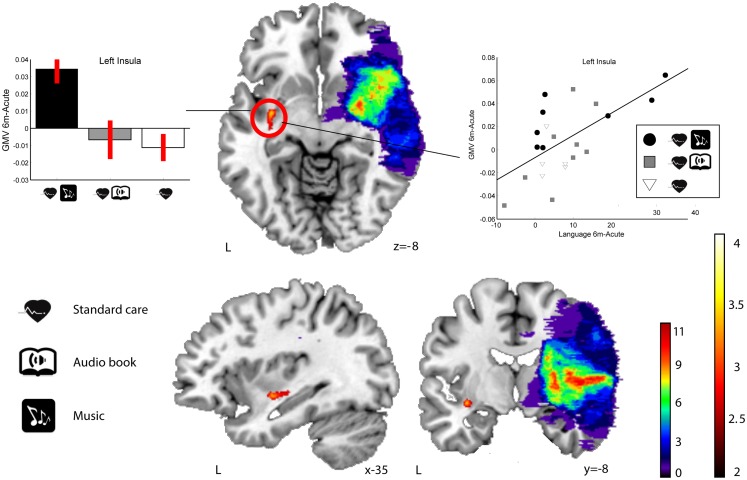
**GMV increases (6-month − acute) in the MG compared to the ABG and CG (RHD patients)**. Blue–green–red: lesion overlap indicating the number of patients showing damage at a particular voxel. Red–yellow: GMV increases for the MG compared to the ABG and CG (Group × Time interaction, MG > ABG, and CG contrast). Bar graphs (left) indicate GMV increases (mean ± SEM) for the only cluster (left insula) showing an interaction effect (white: CG, gray: ABG, black: MG). The scatter plot (right) shows the relationship between GMV increase in the insula cluster and improvement in language skills. Neurological convention is used. Results are shown at *p* < 0.01 (uncorrected) with ≥50 voxels of spatial extent and overlaid over a canonical template with MNI coordinates at the bottom right of each slice. L, left hemisphere.

In RHD patients, there were no significant Group × Time interactions in GMV in any area at the selected threshold (*p* < 0.001 uncorrected). However, when using a slightly more lenient threshold (*p* < 0.005 uncorrected), a single cluster emerged in the left insula (MNI −33 −6 −8; 73 voxels of extent; *t*(22) = 3.36) for the MG > ABG and CG contrast (see Figure 4). Again, no other clusters were found using the reversed contrasts (ABG > CG and MG, CG > MG and ABG) at this same threshold.

There were no significant Time effects or Group × Time interactions in the WMV in LHD or RHD patients.

### Correlation between gray matter changes and behavioral recovery

**Figure 5:**
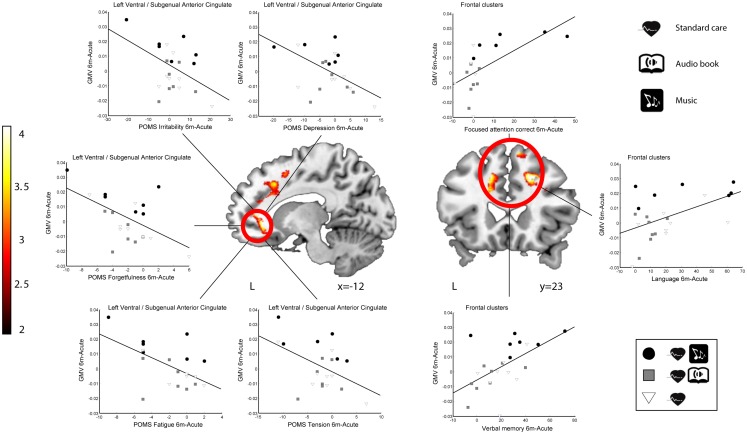
**Correlation between GMV increase and behavioral change (6-month − acute) in LHD patients**. Red–yellow: GMV increases for the MG compared to the ABG and CG (Group × Time interaction). Scatter plots show the relationship between GMV increases and improvements in mood (left ventral/subgenual anterior cingulate cluster) and cognitive variables (three frontal clusters pooled together). Neurological convention is used. Results are shown at *p* < 0.01 (uncorrected) with ≥50 voxels of spatial extent and overlaid over a canonical template with MNI coordinates at the bottom right of each slice (see also Table 6). L, left hemisphere.

In order to determine the functional relevance of the observed GMV increases induced by the music listening intervention, we performed correlation analyses with the longitudinal behavioral data (also 6 months − acute). In LHD patients, the increase in GMV in the identified frontal areas correlated significantly with improvement in verbal memory, language skills, and focused attention (see Table 6 for individual cluster correlations; in Figure 5 the frontal clusters are pooled together for illustrative purposes). Similarly, increase in GMV in the limbic regions (left SACC) was significantly correlated with a decrease in self-reported depression, tension, fatigue, forgetfulness, and irritability and marginally correlated also with a decrease in self-reported confusion. In RHD patients, the GMV increases in the left insula cluster were also found to correlate with the improvement of language skills (*r* = 0.63, *p* < 0.002; Figure 4). There were no other significant correlations.

**Table 6:** **Correlation between GMV increase and behavioral change (6-month − acute) in LHD patients**.

Anatomical area	Behavioral measure	*r*-value	*p*-value
Frontal clusters pooled together	Language	0.51	0.012
	Verbal memory	0.56	0.009
	Focused attention (correct responses)	0.63	0.005
	Focused attention (reaction times)	−0.45	0.063
Right superior frontal gyrus	Language	0.52	0.011
	Verbal memory	0.59	0.004
	Focused attention (correct responses)	0.60	0.009
Left superior frontal gyrus	Language	0.42	0.044
	Verbal memory	0.41	0.050
	Focused attention (reaction times)	−0.61	0.008
Right medial superior frontal gyrus	Focused attention (correct responses)	0.62	0.006
Limbic cluster spooled together	–	–	–
Left ventral/subgenual anterior cingulate gyrus	Depression	−0.61	0.003
	Confusion	−0.41	0.061
	Tension	−0.48	0.029
	Fatigue	−0.65	0.001
	Forgetfulness	−0.55	0.008
	Irritability	−0.45	0.038
Right ventral striatum	–	–	–

*Significant (*p* < 0.05) correlations and linear trends (*p* < 0.1) between the GM volume increases and changes in the summary scores of cognitive tests and the Profile of Mood States (POMS) mood scores [see Särkämö et al. (2008) for details] during follow-up (6 months − acute). Correlations are shown for individual clusters and for groups of clusters within frontal and limbic areas*.

## Discussion

The novel key finding of the present VBM study was that regular music listening during the 6-month post-stroke stage can lead to structural reorganization in the recovering brain. Specifically, compared with patients who listened daily to audio books (ABG) or who did not receive any additional listening material (CG), the patients who listened daily to their own favorite music (MG) showed more increase in GMV from the acute to the 6-month stage in a network of frontolimbic areas, primarily in the healthy contralesional side but also perilesionally. Importantly, the observed GMV increases in this network were directly associated with the behavioral improvement in cognitive functioning and reduction in negative mood shown previously for music listening (Särkämö et al., 2008; Forsblom et al., 2012). The area-specific correlations obtained (attention, memory, and language for frontal areas; mood for limbic regions), the lack of differences in the reversed contrasts (ABG > CG and MG, CG > MG and ABG), and the fact that effects emerge in areas that have previously been found to be closely associated with music processing and cognitive/emotional processing (see below), argue against our results being false positives. Moreover, given that the patient groups were comparable at baseline and the potential effects of other types of rehabilitation (standard stroke rehabilitation) and activities (audio book listening) were controlled for, these findings suggest that a musically enriched environment can be beneficial for acute stroke recovery and that neuroplastic changes in the frontolimbic network may underlie its efficacy.

In the present study, the frontal GMV increases associated with music listening in LHD patients were located in the left and right SFG and the right medial SFG and correlated with the improvement of verbal memory, language skills, and focused attention over the 6-month follow-up. These correlations are well in line with the previous findings of the study showing that music listening enhanced the recovery of verbal memory and focused attention more than audio book listening or standard care both 3 and 6 months post-stroke (Särkämö et al., 2008). In addition, in the original data (*n* = 54), there was also a slight trend toward a group difference in the domain of language [mixed-model ANOVA, Group × Time interaction, *F*(2.4, 51.9) = 2.2, *p* = 0.108)] over the 6-month follow-up, with more improvement in the MG than in the ABG (*p* = 0.096), suggesting that the correlation between GMV and language skills is also meaningful. In previous MEG and fMRI studies on music, the activity of the SFG has been linked to melody discrimination and production (Brown et al., 2006; Lappe et al., 2013), processing the emotional valence of music (Escoffier et al., 2013), and musical episodic memory (Platel et al., 2003). Cognitively, evidence from neuroimaging and lesion studies suggests that the SFG is involved in many domain-general cognitive functions, such as attention and working memory (du Boisgueheneuc et al., 2006; Huang et al., 2013), and together with the dorsal anterior cingulate cortex (DACC) it forms one key component of the *salience/central executive network* (Dosenbach et al., 2008). Interestingly, the DACC/SFG area seems to have a role also in language processing, including internally generated speech (Blank et al., 2003), and its activity has recently been linked also to aphasia recovery (Brownsett et al., 2014). Structural and functional changes in the SFG have also been reported following meditation practice (Kang et al., 2013) and cognitive training (Hoekzema et al., 2010), suggesting that changes in SFG are associated with improved cognition.

In addition to the frontal areas, we also found GMV increases induced by the music listening in LHD patients in two limbic areas: the left SACC and the right VS. Moreover, the GMV increase in the left SACC correlated with the reduction of negative mood (depression, confusion, tension, fatigue, forgetfulness, and irritability) in the POMS questionnaire. Again, this finding is well in line with our previous behavioral results showing that the music listening reduced depression and confusion more than standard care (Särkämö et al., 2008) and was also subjectively associated with better relaxation and positive mood than the audio book listening (Forsblom et al., 2012). Generally, the VS is considered to be a key part of the neural circuitry for reward and pleasure, and its dysfunction is associated with anhedonia, a hallmark symptom of depression (Der-Avakian and Markou, 2012; Eslinger et al., 2012). The nucleus accumbens (NAc) and parts of the caudate nucleus and putamen, the dopaminergic VS, have been strongly implicated in neuroimaging studies as underlying the emotional experience of music (Blood and Zatorre, 2001; Brown et al., 2004; Menon and Levitin, 2005; Koelsch et al., 2006; Mitterschiffthaler et al., 2007; Montag et al., 2011; Salimpoor et al., 2011, 2013). fMRI studies have implicated also the anterior cingulate in processing musical emotions (Brown et al., 2004; Mitterschiffthaler et al., 2007; Green et al., 2008; Janata, 2009; Escoffier et al., 2013), musical preferences (Berns et al., 2010; Kitayama et al., 2013), rhythm and melody perception (Jerde et al., 2011; Lee et al., 2011) and production (Brown et al., 2006; Berkowitz and Ansari, 2008), and singing (Kleber et al., 2007; Zarate and Zatorre, 2008). Generally, the ventral-rostral part of the ACC has a regulatory role in generating emotional responses, and its abnormal functioning has been linked to many psychiatric conditions (Etkin et al., 2011). In depressed patients, the activity of both the ACC and the VS to pleasant music has been found to be reduced compared to healthy controls (Osuch et al., 2009; Aust et al., 2013). In VBM studies, GM loss in the ACC has been linked to impaired recognition of musical emotions in frontotemporal dementia (Omar et al., 2011) and has also been documented as a key neuroanatomical component in the etiology of major depression (Grieve et al., 2013; Lai, 2013).

One possible interpretation for the increase in GMV in the subgenual part of the ACC could be related to the role of this subregion in affective appraisal, integration of emotional and motivational states, self-referential mental processing, and introspective thought (Northoff and Bermpohl, 2004; Vago and Silbersweig, 2012). Interestingly, Greicius et al. (2007) found using PET that the resting-state SACC activity was linked to the *default-mode network* in depressed patients and also correlated with the length of the depressive episode. In a recent fMRI study (Yoshimura et al., in press), the activity of medial prefrontal cortex and ventral ACC during a task of self-referential processing of positive emotional trait words was also observed to increase in depressed patients following cognitive behavioral therapy (CBT), suggesting that these areas are also linked to the amelioration of depression. Thus, given that pleasant and autobiographically salient music can activate the ventral ACC (Janata, 2009) and that music listening was also observed to evoke thoughts and memories about the past and improve mood in our stroke patients (Forsblom et al., 2012), it is possible that positive self-referential emotional processing associated with music listening could be driving the observed structural enhancement of the SACC and the concurrent reduction in depressed mood.

Within the RHD subgroup of patients, we observed more GMV increase in the MG compared to the other groups in a single contralesional (left) cluster in the insula, which also correlated with the recovery of language skills. Since this was seen only using a slightly less stringent statistical threshold (*p* < 0.005 uncorrected) and the RHD–MG listened to the intervention material slightly more often than the RHD–ABG (*p* = 0.076), this result should thus be interpreted with some caution. Although less is known about the specific role of the insula in music or language, current evidence from neuroimaging links it to the affective processing of music (Brown et al., 2004; Menon and Levitin, 2005; Koelsch et al., 2006; Montag et al., 2011; Omar et al., 2011; Trost et al., 2012) and voice (Blasi et al., 2011), musical creativity and improvisation (Brown et al., 2006; Engel and Keller, 2011; Villarreal et al., 2013), and perception of melody (Wehrum et al., 2011) and chords (Koelsch et al., 2005) as well as to verbal functions, especially speech articulation (Ackermann and Riecker, 2010; Price, 2010; Baldo et al., 2011). Overall, there was clearly less music-induced GM reorganization in RHD patients than in LHD patients. One reason for this could be that the lesions in the right hemisphere were, on the average, larger and more extensive than in the left hemisphere (*p* = 0.044). Coupled with the fact that there is a level of right hemisphere dominance for music processing (Zatorre et al., 2002; Tervaniemi and Hugdahl, 2003) and, consequently, the majority of the RHD patients had some degree of amusia (see Särkämö et al., 2009, 2010b), it is possible that the music was not to able to engage the musical brain network in RHD patients to the same degree as in LHD patients. In addition, our sample size was relatively small (26 RHD patients and 23 LHD patients) and there was also considerable variability in the location and size of the lesions (see Figures 1 and 2, top row), which together can affect the sensitivity of the VBM analysis to detect potential volumetric changes over time, especially in the lesioned hemisphere. Thus, larger studies with more homogenous lesion characteristics are called for in the future to verify and extend the current findings.

In general, the exact anatomical nature of the GM and WM changes observed with the VBM method is still not understood very well - in VBM, a change in “volume” essentially refers to a change in GM intensity in the images (not the real volume of neurons, for instance) and is therefore non-specific with respect to the underlying tissue characteristics. According to the current view, the potential mechanisms for GM reorganization include axon sprouting, dendritic branching and synaptogenesis, neurogenesis, changes in glial number and morphology, and angiogenesis (Zatorre et al., 2012). These cellular changes as well as changes in neurotrophic and neural growth factor levels have been documented also in animal studies of post-stroke EE (Biernaskie and Corbett, 2001; Johansson and Belichenko, 2002; Gobbo and O’Mara, 2004; Komitova et al., 2005; Matsumori et al., 2006; Söderström et al., 2009) and auditory EE in healthy developing animals (Engineer et al., 2004; Angelucci et al., 2007; Nichols et al., 2007; Bose et al., 2010), providing experimental support for the enhanced cerebral reorganization induced by the musically enriched recovery environment in the present study.

In conclusion, the present study shows that daily music listening during the first month post-stroke stage can lead to fine-grained structural reorganization (as indicated by increased GMV) in a network of frontolimbic brain areas. Importantly, given that the frontolimbic plastic changes were also directly related to the cognitive and emotional recovery previously shown to be enhanced by music (Särkämö et al., 2008; Forsblom et al., 2012), these findings provide a plausible neuroanatomical correlate for the efficacy of music after stroke. At a more general level, they also provide the first evidence in humans that not only active therapist-led rehabilitation but also environmental enrichment has the potential to shape the structure of the recovering brain.

## Conflict of Interest Statement

The authors declare that the research was conducted in the absence of any commercial or financial relationships that could be construed as a potential conflict of interest.

## Footnotes

^1^http://www.mccauslandcenter.sc.edu/mricro/mricron/index.html

^2^http://www.alivelearn.net/xjview8/
